# Supplementary figures and images for: IFITM1 and IFITM3 cooperate to restrict virus entry in endolysosomes
Source: bioRxiv. 2025 Jun 1:2025.06.01.657267. Preprint. [Version 1] doi: 10.1101/2025.06.01.657267 (PMC12478411; doi:10.1101/2025.06.01.657267)

Supplemental Figure 1

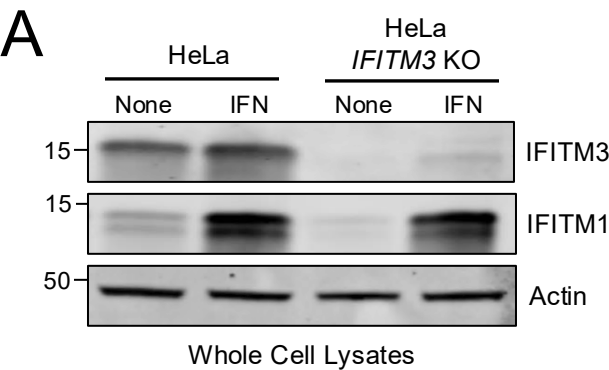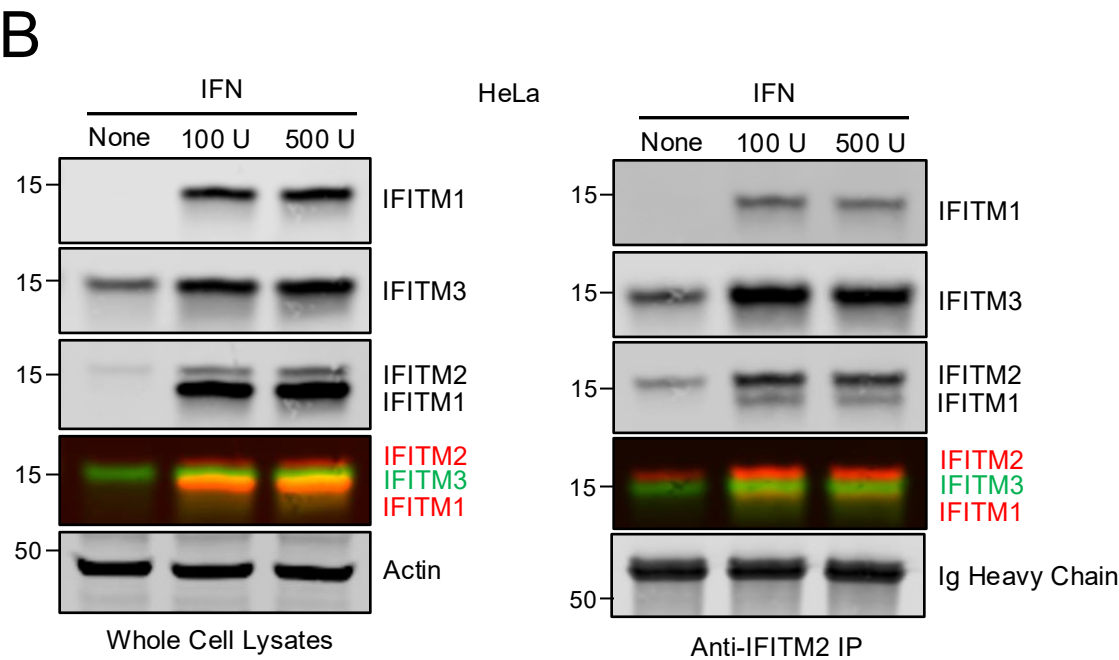

Supplement: Supplement 1 — Supplemental Figure 1: (A) HeLa and HeLa IFITM3 knockout cells were untreated or treated with 100 units IFNb for 18 hours and lysed. Whole cell lysates were subjected to SDS-PAGE and immunoblotting with anti-IFITM3, anti-IFITM1, and anti-Actin (used as loading control). (B) Left: HeLa cells were untreated or treated with 100 units or 500 units IFNb for 18 hours and lysed. Whole cell lysates were subjected to SDS-PAGE and immunoblotting with anti-IFITM1, anti-IFITM3, anti-IFITM2, and anti-Actin (used as loading control). Right: IFITM2 was immunoprecipitated with anti-IFITM2 and IP fractions were subjected to SDS-PAGE and immunoblotting with anti-IFITM1, anti-IFITM3, and anti-IFITM2 (immunoglobulin heavy chain was used as loading control). Numbers and tick marks left of blots indicate position and size (in kilodaltons) of protein standard in ladder. [file media-1.pdf]
